# Methodological limitations and confounders in dermal toxicity evaluation of aqueous test substance by OECD technical guidelines 402, 410: our experience of testing ethanol based hand sanitizer

**DOI:** 10.1080/07853890.2025.2491664

**Published:** 2025-04-17

**Authors:** Rajendra Palkhade, Deepali Sammal, Jitendra Parmar, Snehal Chavhan

**Affiliations:** a Biological Sciences Division, ICMR-NIOH, Ahmedabad, Gujarat, India; b NFSU, Gandhinagar, Gujarat, India; c Animal House, ICMR-NIOH, Ahmedabad, Gujarat, India; d Health Sciences Division, ICMR-NIOH, Ahmedabad, Gujarat, India

**Keywords:** OECD TG 402, OECD TG 410, acute dermal toxicity, subacute dermal toxicity, ethanol based hand sanitizer

## Abstract

**Background:**

In view of overzealous use of Alcohol based hand sanitizer (ABHS) during COVID-19 pandemic and associated alarming rise in the cases of hand eczema and dermatitis around the world. We conducted an *in vivo* dermal toxicity with objective of exploring the acute and subacute effects of ethanol based hand sanitizer (EBHS) on Sprague Dawley rats.

**Aims:**

To evaluate acute and subacute dermal toxicity due to ethanol based hand sanitizer (EBHS) on Sprague Dawley rats.

**Methods:**

In first phase, following Organisation for Economic Co-operation and Development (OECD) Technical Guidelines (TG) 402, we conducted acute dermal toxicity study with two rats and EBHS containing 72.34% ethanol. In second phase, sub-acute dermal toxicity study was conducted, following OECD TG 410 with five groups of rats (10 animals of either sexes in each group) at various doses.

**Results:**

In both the studies, no erythema, oedema, and eschar was observed. Although no clinical signs of toxicity were observed in both the studies, one death was encountered in subacute study. Macroscopically skin was normal; however, microscopic changes such as hyperkeratosis, parakeratosis, erosion, and extracellular oedema in epidermis and diffuse inflammatory cell infiltration in dermis was observed, suggestive of spongiotic dermatitis and ‘clinic-pathological discordance’. However, attributing this changes to ethanol is difficult due to methodological limitations and confounders.

**Conclusion:**

In both the studies, ethanol based hand sanitizer (EBHS) was found to be non-irritant with LD_50_ of > 2000 mg/kg and classified as Class 5/Unclassified according to GHS classification. Although, spongiotic changes were observed, methodological limitation of absence of control group in TG 402 and confounding effect of water and occlusion in all the animals/groups in both studies prevented us to attribute it to ethanol.

## PLAIN TEXT SUMMARY

In COVID19 pandemic, an emergency measure of hand hygiene, i.e. Alcohol -based hand sanitizers (ABHS); Ethanol- based hand sanitizer (EBHS) in Indian context, has been used overzealously for prolonged period. This has led to rise in cases of skin changes in hands, which was reported by many dermatologists around the world. We tested a commonly used EBHS by applying it to rat skin on a single dose in acute dermal toxicity evaluation and repeated dose in subacute dermal toxicity evaluation. Although, we did not find gross skin changes in both the studies, microscopic changes in the epidermis and dermis suggestive of spongiotic dermatitis were observed. However, due to absence of control group in acute study, and presence of water in test substance along with occlusion of skin which independently can give rise to such microscopic picture, we could not implicate ethanol for microscopic changes in skin. Such situation demands modification in methodology in OECD TG 402 and 410 while testing an aqueous test substance. Also, simultaneous conduct of both acute and subacute studies is recommended for testing aqueous test substance because acute study conducted in isolation without control group may give erroneous results. In view of such inbuilt methodological limitations and confounder, use of newer *in vitro*, and molecular techniques is recommended for reliable toxicological evaluation of aqueous base substance.

## Introduction

COVID-19 compelled World Health Organization (WHO) and United Nations Children’s Fund (UNICEF) to adopt emergency use of alcohol based hand sanitizer (ABHS) in absence of soap and water to prevent disease transmission [1]. India also followed the suit and the Ministry of Consumer Affairs recommended the use ethanol based hand sanitizer (EBHS) for emergency use in its order letter no. 1(2)/2020-sp-1 available on website [2]. Recommendation to use alcohol based hand sanitizers (ABHS) for hand hygiene has led to its overzealous use for extended period [3,4]. This is also supported by the report of 1400% increase of ethanol production during COVID-19 pandemic [5]. Among consumers of ABHS, health care workers are specifically vulnerable to long term impact of these alcohol based sanitizers due to repeated use in their occupational settings [6]. During the pandemic, American contact dermatitis society anticipated a rise in cases of allergic contact dermatitis (ACD) and irritant contact dermatitis (ICD) [7] which became a reality, as evidenced by numerous case reports, case series and epidemiological studies that have shown deleterious acute impact of ABHS on skin in COVID19 pandemic [8–14]. A case reports also suggested that long term use of ABHS can induce neurotoxicity as well as cardiotoxicity [15].

With this background, we conducted an acute and subacute dermal toxicity study with objective of evaluating the acute and sub-acute dermal toxicity of ethanol based hand sanitizers (EBHS) in Sprague Dawley (SD) rats.

## Methods

After seeking approval from the 48th Scientific Advisory Committee (SAC) (Agenda 6.1.7, IM/SNC/21-22/07) and the Institutional Animal Ethics Committee (IAEC) 25th IAEC meeting agenda No. 05, held on 29 November 2021 (IAEC/NIOH/2021-22/25/03/R) of ICMR-National Institute of Occupational Health (ICMR-NIOH), Ahmedabad, Gujarat, India, the study was carried out as per OECD test guideline 402(TG 402) [16] adopted in 2017 and (TG 410) [17] and used Arrive guidelines 2.0 [18] for reporting the findings.

As per the principals described in TG 402, an acute dermal toxicity study is conducted in two steps: (i) Range-finding study and (ii) Main study. The range-finding study serves the purpose of finding an initial dose for main study in absence of data related to LD_50_ of the test chemical.

However, information regarding dermal LD_50_ of ethanol was available from European Chemical Agency (ECHA), which was >15,800 mg/kg, i.e. (15.8 gm/kg) [19]. In view of animal welfare, we avoided using animals for range-finding study as prescribed in OECD TG 402 and saved 4–5 animals.

Considering the high dermal LD_50_ value, we conducted the study using limit dose of 2000 mg/kg, which was prescribed in earlier versions of OECD Technical Guidelines (TG 402) 1987 [20] with expectation to produce clear signs of toxicity without causing severe toxic effects or mortality.

**Figure 1.**
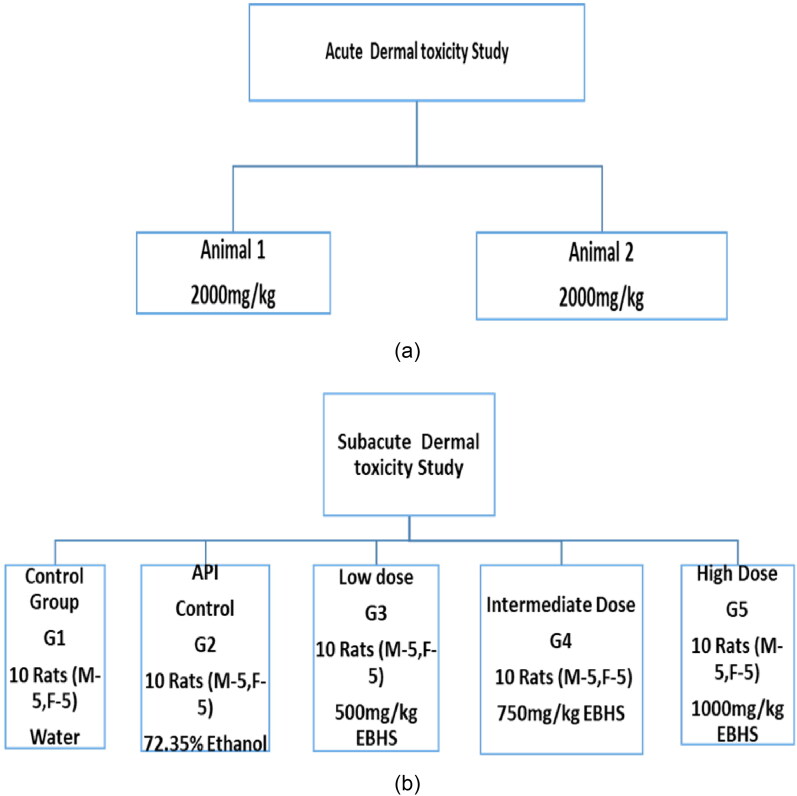
(a) Framework of acute dermal toxicity study. (b) Framework of Sub-acute dermal toxicity study.

In case of subacute dermal toxicity study, the test substance was applied daily to the skin in graduated doses (Figure 1a and 1b) to five groups of SD rats, one dose per group, for a period of 28 days as prescribed in OECD TG 410. During the period of application, the animals were observed daily for morbidity and mortality, if any. Animals which died during the test were necropsied, and at the termination of the test the surviving animals were sacrificed and necropsied.

### Test chemical

We selected an EBHS that was widely used and whose Material Safety Data (MSD) was available in public domain. We sourced it from local medical shop.

### Chemical composition

Test EBHS contained – Ethanol-72.34% Denatured Alcohol w/w, Water, PEG/PPG-17/6 copolymer, Propylene Glycol, Acrylates cross polymer, Tetrahydroxypropyl­ethylenediamine, Perfume. Mfg. date −12/21, Expiry date − 11/24.

### Animal characteristics

Species—Rats. Strain—Sprague Dawley (SD). Sex—Male and Female. Healthy Sprague Dawley rats were procured from Zydus Research Centre, Changodhar, Ahmedabad Gujarat (CCSEA, New Delhi registered breeder). Females were nulliparous and non-pregnant. Each animal, at the commencement of its dosing, was a young adult (8–10 weeks old). In acute study, animals were weighing 200–300 grams whereas in subacute study animal weight of female was in the range of 250–300 grams and males were weighing between 350–400 grams.

### Housing and augmenting environments

Experimental animal room temperature was kept between 18–25 °C. Relative humidity (RH) of 50–60%was maintained. Artificial lighting was used with 12-h light and dark cycle. Common lab diets were fed accompanied by an ad.lib supply of RO drinking water.

### Preparation of animals

Study animals were acclimatized to the laboratory conditions for 7 days before actual experiment, during which they were group-caged for welfare reasons. At the end of acclimatization period, animals were grouped randomly and marked with picric acid to provide individual identification avoiding the back/flank of animal. Twenty-four hours before the application of dose, approximately 10% of the total body surface area (TBSA) was closely trimmed to remove the fur from the dorsal/flank area of the test animals. No anaesthetics were used to aid in handling animals. Animals were handled by the trained technician under the supervision of study veterinarian to avoid stress to animals. Intactness of skin was ensured by avoiding abrasion to the skin.

### Number of animals

Two female SD rats were used in acute dermal toxicity evaluation as per OECD TG 402. According to OECD TG 410, 50 animals were used, divided in five groups with 10 animals (1:1 sex ratio) at various doses with API and normal saline control group in subacute dermal toxicity evaluation.

### Dosage

Acute study was conducted at limit dose 2000 mg/kg, i.e. 2 gm/kg body weight. While graduated doses such 500 mg/kg, 750 mg/kg, and 1000 mg/kg were taken for subacute study. Dosage were calculated after adjusting for specific gravity of 0.9 gm/l and considering ethanol content of 72.34% w/w as follows:

Volume adjustment factor = Specific gravity of water/Specific gravity of liquid preparation = 1.00000/0.9 gm/*L* = 1.1111 (approximately)

Required volume at water’s specific gravity = Original volume * Volume adjustment factor = 1 mL * 1.1111 = 1.11 mL (approximately). So instead of 1 ml a dose of 1.1 ml was taken and accordingly dose was calculated as per animal’s weight.

### Administration of doses

The EBHS was applied as uniformly as possible over the exposed area of dorsal/flank skin (i.e. 10% of TBSA) so that it was covered with as thin and uniform film of EBHS. Continuous contact of EBHS with skin was ensured by covering it with a porous gauze dressing (Glosurge^TM^, Gauuze swab B.P. type −17, 5 cm*5 cm*16 ply) and wrapping it with semi occlusive bandage (Wilson^TM^, Adhasive tape U.S.P, 5 cm*8 mtr) to stabilize the gauze dressing. This measure avoided ingestion of EBHS by the animals throughout exposure period avoiding use of restrainers. During the exposure period, animals were caged individually in order to avoid oral ingestion of the EBHS by other animals in the cage. As we were testing liquid test chemical, we used it undiluted as per guidelines. At the end of the exposure period, the EBHS residues were removed using cotton swab soaked in water. The animals were returned to their individual cages and observed.

### Exposure frequency

In Acute dermal toxicity study, animal exposure was done only once, on 1st day. While in subacute dermal toxicity study, animals were exposed daily for 6 hours, 5 days a week, for 28 days.

### Observations

The animals were observed periodically for toxicological signs, physical, and behavioural changes at dosing, once every 30 min in first hour, then hourly monitoring was done for 24 hours, and daily thereafter, for a total of 14 days in acute, and 28 days in subacute study. Body weight was recorded every day until the day of termination of study.

### Skin histology

Dorsal skin was sampled on the day of necropsy, fixed in 10% formalin solution ,and embedded in paraffin wax. H&E (Himedia) staining was performed on skin sections. Histopathological findings were evaluated as individual scores graded as follows – No abnormality: 0, Slight: +1, Mild: +2, Moderate: +3 for each pathological changes, including hypertrophy, hyperkeratosis, parakeratosis, erosion, inflammatory cell infiltration, extracellular oedema, and ulcer, as described in Modified Hirasawa Scoring system adopted by Watanabe et al. [21] The findings were visualized at 10x and 40 x magnification.

## Results

### Draize’s skin irritation test [22]- observations

Draize’s skin irritation test was uneventful in both acute and subacute study. Oedema, Erythema, and Eschar was not observed in 72 hours of observation period.

### Cage side observation

In observation period of 14 days and 28 days respectively in acute and subacute study, no fur changes, ocular changes, sleep, convulsions, tremors, diarrhoea, lethargy, salivation, coma were observed in any animal. Animals showed normal behaviour, hair growth, water intake, urine output, and stool. Furthermore, eyes, mouth, nose, and ears were devoid of any abnormal secretions. Although, acute dermal toxicity was devoid of any mortality, one death was observed in intermediate dose group (G 4–750 mg/kg) in 4th week of subacute dermal toxicity study. Immediate necropsy was performed on deceased animal and the findings were suggestive of death due to internal haemorrhage’s (Lung, Kidney, Intestine, Urinary bladder) unrelated to exposure.

**Figure 2.**
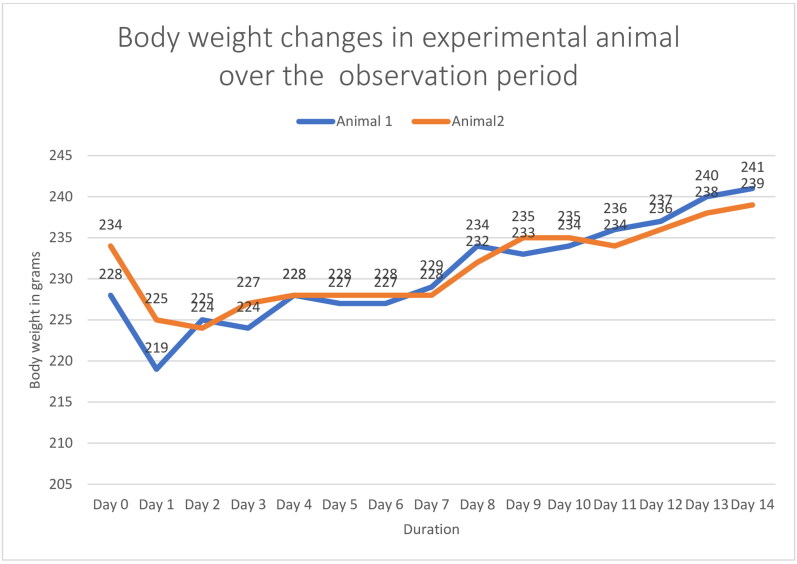
Showing body weight changes observed during acute dermal toxicity evaluation.

Body Weight Changes—In acute study, an unexplained decrease in body weight of both the animals was seen in initial 1–3 days, which could be due to application of semi occlusive bandage. (Figure 2) However, the animals regained weight in subsequent study duration.

**Figure 3.**
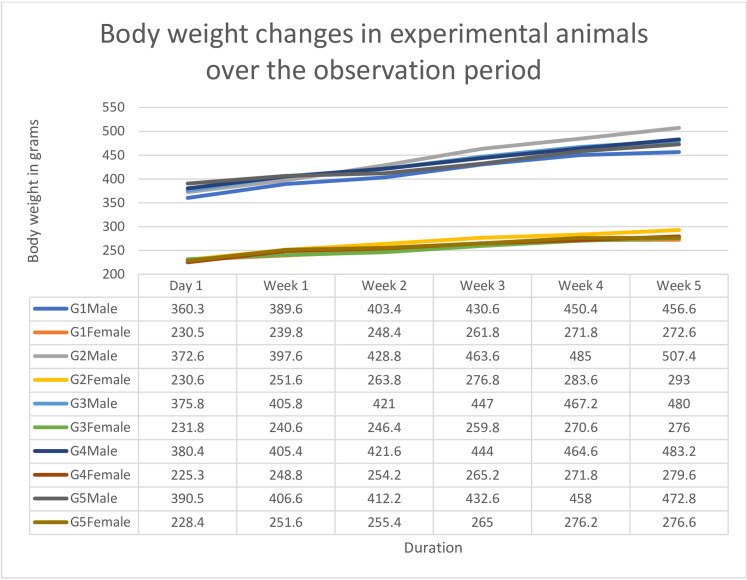
Showing body weight changes observed during sub-acute dermal toxicity evaluation.

In subacute study, we did not encounter any decrease in body weight of animals throughout the experiment (Figure 3).

#### Necropsy findings

Macroscopically, the visceral organs appeared normal without any gross abnormality. The volume, colour, and texture of visceral organs were comparable with control group (subacute study). In case of acute toxicity study, no abnormality was detected.

### Skin histopathology

**Figure 4.**
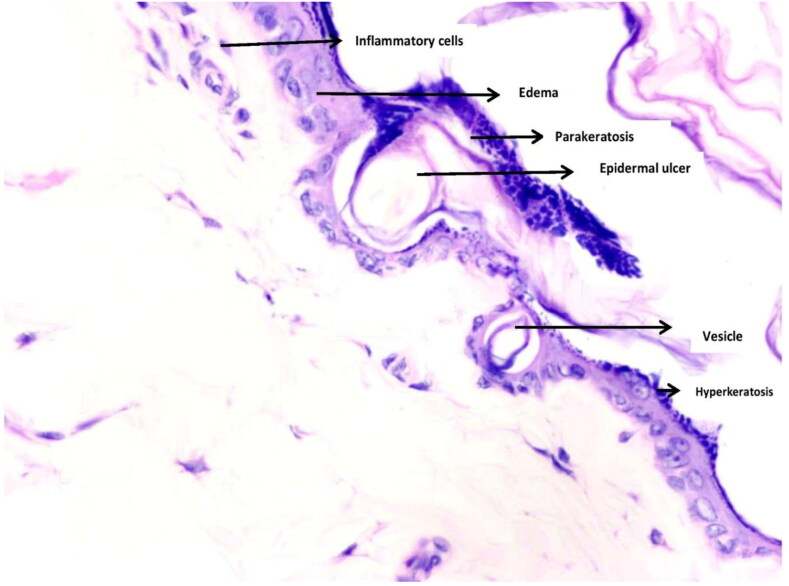
Showing histological findings of hyperkeratosis, parakeratosis, erosion/ulcer, extracellular oedema, and vesiculations in epidermis with diffuse inflammatory cell infiltration in dermis and normal Sub cutis observed in both the studies.

In both the studies and across all the study groups, skin histopathology showed changes in epidermis, such as hyperkeratosis, parakeratosis, erosion/ulcer, extracellular oedema, and vesiculations. In dermis, diffuse inflammatory cell infiltrates were seen. Subcutis was within normal limits. Such features are typically seen in spongiotic dermatitis (Figure 4).

Further, there were no significant differences in the histological findings of epidermis and dermis across the animals and groups according to modified Hirasawa Scoring System adopted by Watanabe et al. (Tables 1 and 2).

**Table 1.** Showing grades of pathological changes observed in acute dermal toxicity evaluation according to modified Hirasawa scoring system adopted by Watanabe et al. [21].

Anatomical region Pathological	Group	Animal 1				Animal 2			
	Grade	–	1+	2+	3+	–	1+	2+	3+
	change								
Epidermis	Hypertrophy	0	1	0	0	0	1	0	0
	Hyperkeratosis	0	1	0	0	0	1	0	0
	Parakeratosis	0	0	0	0	0	1	0	0
	Erosion	0	0	0	0	0	1	0	0
	Inflammatory cell infiltration	0	0	0	0	0	0	0	0
	Extracellular oedema/vesicles	0	1	0	0	0	1	0	0
Corium/dermis	Ulcer	0	0	0	0	0	0	0	0
	Inflammatory cell infiltration	0	1	0	0	0	1	0	0
Subcutis	Inflammatory cell infiltration	0	0	0	0	0	0	0	0

Grade of histological finding; 0: no abnormality, 1+: slight, 2+: mild, 3+: moderate.

**Table 2.** Showing grades of pathological changes observed in sub-acute dermal toxicity evaluation according to modified Hirasawa scoring system adopted by Watanabe et al. [21].

	Group	G1 *n* = 10				G2 *n* = 10				G3 *n* = 10				G4 *n* = 10				G5 *n* = 10			
Anatomical region	Grades	0	+1	+2	+3	0	+1	+2	+3	0	+1	+2	+3	0	+1	+2	+3	0	+1	+2	+3
	Pathological change																				
Epidermis	Hypertrophy	0	8	1	1	0	6	2	2	0	7	1	2	0	8	1	1	0	7	0	3
	Hyperkeratosis	1	7	1	1	0	8	1	1	0	8	1	1	0	7	1	2	0	7	2	1
	Parakeratosis	3	5	1	1	2	6	1	1	0	8	1	1	0	8	1	1	0	8	1	1
	Erosion	5	3	1	1	6	1	2	1	6	1	2	1	5	3	1	1	5	3	1	1
	Inflammatory cell infiltration	0	0	0	0	0	0	0	0	0	0	0	0	0	0	0	0	0	0	0	0
	Extracellular oedema/vesicles	6	3	0	1	5	2	1	2	7	1	2	0	6	2	2	0	5	4	0	1
Corium/Dermis	Ulcer	0	0	0	0	0	0	0	0	0	0	0	0	0	0	0	0	0	0	0	0
	Inflammatory Cell Infiltration	0	9	1	0	0	8	2	0	0	9	1	0	0	7	1	1	0	7	3	0
Subcutis	Inflammatory Cell Infiltration	0	0	0	0	0	0	0	0	0	0	0	0	0	0	0	0	0	0	0	0

Grade of histological finding; 0: no abnormality, 1+: slight, 2+: mild, 3+: moderate.

## Discussion

In present study of toxicological evaluation of acute and subacute dermal toxicity of EBHS, EBHS was found to be nonirritant according to Draize’s criteria, on skin of rats. Although, no clinical signs of toxicity were observed in both the studies, one death was encountered in subacute study. Macroscopically skin was normal; however, microscopic changes such as hyperkeratosis, parakeratosis, erosion, and extracellular oedema in epidermal layer and diffuse inflammatory cell infiltration in dermis was observed in both the studies suggestive of spongiotic dermatitis.

Historically, in 1975 and 1985, CDC came up with guidelines on hand washing practices in health care setting. Initially, only in emergency conditions, when sink and water would be unavailable, was the use of alcohol-based solutions recommended. In addition, 1995 and 1996, the Healthcare Infection Control Practices Advisory Committee (HICPAC) recommended for use of either antimicrobial soap or alcohol-based solutions while caring for patients infected with multidrug-resistant (MDR) pathogens [23]. However, in COVID19 pandemic, these HCW specific recommendations were made applicable for masses for prevention of transmission, which lead to overzealous and prolonged use of ABHS.

Rundle et al. reported that the American Contact Dermatitis Society anticipated an increase in both irritant contact and allergic contact hand dermatitis in COVID-19, considering stringent hand hygiene practices followed during COVID times [7]. The anticipation turned into reality when dermatologists around the world started publishing Clinical (case reports/case series) and epidemiological studies of dermatitis wherein dermatitis was attributed to exposure to ABHS.

Case reports and case series by Pope and Ousley [9], Inder and Kumar [10], Kar et al. [14] highlighted the noxious nature of ABHS.

The evidence was further strengthened by epidemiological studies done by Alsaidan et al. [8] and Jindal et al. [13] where they reported that approximately 34% of general population was affected by hand dermatitis due to excessive use of ABHS.

Among the affected population, occupational group of health care workers was highly susceptible and the same was reported by Lan et al. [24], Saha et al. [25], Toplu et al. [26], Guertler et al. [27], Chernyshov and Kolodzinska [28].

Above mentioned studies formed a rational of conducting a dermal toxicity of EBHS. However, in our study, we did not encounter any macroscopic change in skin of rat which is contradictory to the findings of above mentioned studies. A possible explanation could be that in acute dermal toxicity study, one-time exposure was given as opposed to requirement of repeated hand washing in hospital setting due to strict adherence to hand hygiene practices during COVID19. Although, subacute dermal toxicity exposure does resemble with repeated exposure, we believe that the frequency of hand hygiene would still be higher in real life situation as opposed to once a day dosing of experimental setting.

Literature also exists where some researchers have advocated for the use of ABHS, saying that dermatitis like reactions are rare phenomena and, if at all occurs, are short lived and not associated with ABHS use. Researchers recommending use of ABHS use are Jeannie et al. [11], Graham et al. [29], Yan et al. [30], Luise and Borch et al. [31] Similar recommendations in favour of ABHS use was given by Lotfinejad and Peters [32] and Moore et al. [33].

Although our gross skin findings are in consonance with above mentioned studies which are in favour of ABHS, none of the authors have clearly refuted occurrence of dermatitis due to ABHS. Despite occurrence of skin changes in their respective studies, they went on to recommend the use of ABHS, considering the benefits in pandemic situation. However, microscopic findings of our study points towards pathological spongiotic changes in epidermis and dermis, indicative of dermatitis, which could be an irritant contact dermatitis at higher dose in acute dermal toxicity evaluation or allergic contact dermatitis in subacute dermal toxicity evaluation due to repeated exposure.

Microscopic features of our study are in consonance with human studies done by Ophaswongse and Maibach who reported ethanol itself can act as an allergic compound in both immediate as well as delayed hypersensitivity reactions and concluded that ethanol induced allergic contact dermatitis could be more frequently seen in clinical practice but less frequently reported in literature because it is possibly overlooked or misdiagnosed [6].

Work of Lübbe and colleagues [34,35], Okazawa et al. [36], Wilkins and Fortner [37] and Das et al. [38] points towards irritant potential of alcohol and resultant irritant contact dermatitis.

Bhatia et al. reported that ethanol can act as an allergen as well as irritant, and occasionally both ICD and ACD can coexist in a single patient [39].

Talking about *in vivo* studies, our findings are similar to results of Ahn et al. who evaluated the irritation potential of alcohol-based hand sanitizers containing *Aloe vera* Linn. and clove extract by conducting the Draize’s skin test in New Zealand White rabbits where they concluded that the test substance was non-irritating to rabbit skin. However, concentration of ethanol in their preparation was 59% [40] whereas in our study it was 72.34%.

### Although histological findings of our study points towards alcohol induced dermatitis, can we conclusively attribute it to ethanol present in EBHS?

The answer is **‘NO’** due to methodological limitations and confounders that are inherently present in OECD TG 402 and TG 410.

### Methodological challenges in OECD 402

#### i. Absence of control groups

Recent OECD TG 402 adopted in 2017, prescribing methodology to conduct acute dermal toxicity studies recommends use of only two animals in the main study, which we feel is inadequate to draw conclusion. A control group is required for attributing the finding to exposure. Addition of i. Exposure control ii. Vehicle control iii. API control can give meaning to the result and power of attributing the result to exposure which was clearly lacking in our study due to strict adherence to prescribed OECD Guidelines. We observed that its due to same constraint, acute dermal toxicity studies even after publication of 2017 modification, were done with modifications of adding control groups as done by Wanjari et al. [41] who used 20 animals in 2 groups with either sex for evaluation of Laghu vishgarbha taila, where one group acted as a vehicle control, Espinosa-Espinosa et al. [42] also conducted an acute dermal toxicity study of methanolic extract of M. indica employing 3 groups consisting of 2 control groups (Vehicle and exposure control) with 3 animals in each group, Saleem et al. [43] used 5 groups of animals consisting of 2 control groups (exposure and vehicle control) with 5 animals in each group for acute dermal toxicity study of M. neglecta crude methanolic extract, Mishra et al. [44] conducted their acute dermal toxicity evaluation of Calendula officinalis (CO) essential oil using 24 animals divided in 4 groups with one group as an exposure control.

The only study which followed OECD TG 402 in meticulous manner was conducted by Seol et al. [45] in their evaluation of titanium dioxide TiO2 (P-25 and GST). They used five animals in total, three were used for range-finding study and two animals for main study. Our methodology resembles the methodology adopted by them. However, histology data of their study was not included in their results which could have thrown more light on microscopic findings encountered by us.

#### ii. Clinico-pathological discordance

The absence of clear clinical signs of dermatitis in cage side observations, and normal skin at necropsy deter us to conclusively attribute histological findings to EBHS exposure. This is a commonly seen phenomena in medicine, known as ‘clinico-pathological discordance’ which was nicely explained by Farzania et al. in their study, where they evaluated concordance of the clinical and histopathological diagnoses of oral and maxillofacial biopsy specimens. They reported varying degree of concordance between clinical and histopathological findings and concluded that 100% concordance was unachievable in real life setting [46]. Other studies carried out by Warner et al. [47] Jolly and Swan [48], Lindberg and Forslind [49], Lindberg et al. [50], Mikulowska [51,52], Kligman [53]. also underscores similar view even though their test exposure/chemicals might be different.

Although, methodological limitation of lack of control group was negated in subacute dermal toxicity TG 410, we are challenged by a confounder. Similar confounders were also inherently present in methodology of acute dermal toxicity evaluation discussed further.

### Water as a confounder

Although, considered to be no harmful, the innocuous nature of water has been challenged by researchers like Warner et al. [46] who have conclusively shown that water can itself act as an irritant to skin. Using transmission and cryo-scanning electron microscopy, they confirmed, that the water exposure of extended period leads to extensive disruption of stratum corneum intercellular lipid lamellae. They showed exposure to water for 4 or 24 hours results in a 3 and 4-fold expansion of the stratum corneum thickness, respectively. Corneocytes swell uniformly and hydration induces large pools of water in the intercellular space. Other studies illustrating the disruptive effect of water were done by Renshaw [54], Suskind and Ishihara [55], Possick [56], Halkier-Sorensen et al. [57], Meding [58]. Our results are in congruence with above mentioned studies, where we also observed similar changes in skin microscopy in the acute dermal toxicity study due to 24 h. exposure with EBHS. With such long standing exposure of the skin, over hydration is very likely which might have reflected as a dermatitis like changes due to presence of water as a constituent in EBHS. In subacute dermal toxicity study also, we observed dermatitis like features across all the groups including control due to the presence of water in EBHS. Previous studies done by Jolly and Swan [48], Lindberg and Forslind [49], Lindberg et al. [50], Mikulowska [51,52], Kligman [53] have shown that continuous exposure to water for 6 to 48 hours have resulted in keratinocyte vacuolization and changes in langerhans and mononuclear cells which resembles our findings. It is pertinent to state here that, due to presence of water in aqueous test solutions, their evaluations remain inconclusive following the OECD TG 402, 410. It may be because of same reason acute and subacute dermal toxicity evaluation published recently were mainly pertaining to oil based preparations (as discussed above in section of methodological limitations).

### Occlusion as a confounder

OECD technical guidelines 402 and 410 prescribe use of porous gauze dressing covered by non-irritating tape to keep test chemical in contact with skin, further covering is also prescribed for retention of gauze dressing and prevention of ingestion of test chemical by animal, which however results in occlusion of skin. Fluhr et al. [59] evaluated barrier damage caused by occlusion of (24–96 hours) on the forearm and did not find significant change in hydration and water holding capacity, which contradicts our findings. However, in study carried out by Friebe et al. [60] they investigated the time course of effects of occlusion by epicutaneous patches with 2 doses of sodium lauryl sulphate (SLS), water, and an empty test chamber (control) applied on the volar forearm for different time intervals (12, 24, 48 hours). The effect was assessed through TEWL (Trans epidermal Water Loss) which is a marker of Skin barrier function. They observed immediate and prolonged increase in TEWL values in patch testing with SLS as well as with water and empty patches, which was significant enough to conclude that irritation can be caused by occlusion itself and occlusion in patch testing causes changes in epidermal function that were indistinguishable from the effect of the irritant itself. Studies carried out by Agner and Serup [61], Löffler et al. [62], Tupker et al. [63] also confirm the same view. Researchers like Ryatt et al. [64] mentioned that short-time (30 min) occlusion can result in significantly increased penetration and horny layer water content which is in consonance with present study findings (acute dermal toxicity study). Fregmen et al. [65] have shown that with 24 hours occlusion, the relative water content in stratum corneum can be increased significantly from 53% without occlusion to 59% with occlusion. Matsumua et al. [66] have shown that 24 hours occlusion can induce morphological changes on the surface, and deepening skin furrows. In case of subacute dermal toxicity, where occlusion was done for 8 hours a day consecutively for 28 days (5 days in a week), dermatitis like picture on microscopy was observed. Similar results were observed by researchers like Kligman [67] who reported typical signs of inflammation such as vasodilation, perivenular lymphocytic infiltration, oedema, mast cell degranulation, and proliferation of fibroblasts after applying water-soaked patches under occlusion on normal skin for up to 1–3 weeks, which is similar to our results. Skin damage in subacute evaluation looks obvious because of prolonged exposure as opposed to acute evaluation. Fartasch et al. [68] studied responses to daily water exposure, and occlusion over a period of 7 days in 73 volunteers. They reported that previously occluded, and the water-exposed sites induced higher TEWL and clinical scores in a time-dependent fashion as compared to the control areas, with more pronounced reactions in the water-exposed sites than in the occluded sites. The findings suggest that, after water exposure or the use of occlusive gloves, the skin seems to be more prone to react to stress and inducing higher susceptibility to irritants.

## Conclusion

We finally conclude that after stringently adhering to OECD TG 402 and 410 for acute and subacute dermal toxicity evaluation respectively, the ethanol based hand sanitizer (EBHS) was found to be nonirritant on skin of rats. LD_50_ of EBHS was >2000 mg/kg, and thus, it can be classified as Class 5/Unclassified, according to GHS classification. Although, microscopic changes were suggestive of sponigiotic dermatitis in both the studies, presence of similar microscopic picture in control group animal’s points towards confounding effect of water and occlusion, which was encountered in exposure of all the animals in both studies. Further, methodological limitation of absence of control group/animal in acute dermal toxicity study makes it difficult to attribute microscopic changes to ethanol present in EBHS. Clinico-pathological discordance also further complicated the study. In such scenario, we conclude that, aqueous base solutions require a different methodological approach for dermal toxicity studies, and with conventional methodology results can be inconclusive.

## Data Availability

The data and materials supporting the results or analyses presented in the paper will available upon reasonable request to corresponding author through email at snchavan14787@gmail.com.
